# Decrease of MtDNA copy number affects mitochondrial function and involves in the pathological consequences of ischaemic stroke

**DOI:** 10.1111/jcmm.17262

**Published:** 2022-07-05

**Authors:** Zhaojing Zhang, Dongzhi Yang, Baixue Zhou, Yingying Luan, Qihui Yao, Yang Liu, Shangdong Yang, Jing Jia, Yan Xu, Xiaoshuai Bie, Yuanli Wang, Zhihao Li, Aifan Li, Hong Zheng, Ying He

**Affiliations:** ^1^ Department of Medical Genetics & Cell Biology School of Basic Medical Sciences Zhengzhou University Zhengzhou China; ^2^ School of Life Sciences Zhengzhou University Zhengzhou China; ^3^ Department of Pathology The Second Affiliated Hospital of Zhengzhou University Zhengzhou University Zhengzhou China; ^4^ Department of Neurology The First People's Hospital of Zhengzhou Zhengzhou China

**Keywords:** ischaemic stroke, mitochondrial function, mtDNA, *TFAM*

## Abstract

The mtDNA copy number can affect the function of mitochondria and play an important role in the development of diseases. However, there are few studies on the mechanism of mtDNA copy number variation and its effects in IS. The specific mechanism of mtDNA copy number variation is still unclear. In this study, mtDNA copy number of 101 IS patients and 101 normal controls were detected by qRT‐PCR, the effect of D‐loop variation on mtDNA copy number of IS patients was explored. Then, a *TFAM* gene KD‐OE PC12 cell model was constructed to explore the effect of mtDNA copy number variation on mitochondrial function. The results showed that the mtDNA copy number level of the IS group was significantly lower than that of the normal control group (*p *< 0.05). The relative expression of *TFAM* gene mRNA in the cells of the OGD/R treatment group was significantly lower than that of the control group (*p *< 0.05). In addition, after *TFAM* gene knockdown and over‐expression plasmids were transfected into HEK 293T cells, mtDNA copy number and ATP production level of Sh‐TFAM transfection group was significantly decreased (*p *< 0.05), while mtDNA copy number and ATP production level of OE‐TFAM transfected group were significantly higher than that of blank control group and OE‐ctrl negative control group (*p *< 0.01). Our study demonstrated that mitochondrial D‐loop mutation and *TFAM* gene dysfunction can cause the decrease of mtDNA copy number, thus affecting the mitochondrial metabolism and function of nerve cells, participating in the pathological damage mechanism of IS.

## 1 INTRODUCTION

Ischaemic stroke (IS) refers to the disease of neurological dysfunction due to the stenosis or occlusion of cerebral feeding artery (carotid artery and vertebral artery) and insufficient cerebral blood supply.^1^, ^2^ The pathological mechanism of IS is extremely complex. After cerebral ischaemia, nerve cells will produce a series of cascade damage, such as oxidative stress, energy disorder, excitotoxicity, acidosis, inflammatory reaction, calcium homeostasis imbalance and apoptosis, which eventually lead to central nervous system dysfunction.^3^, ^4^

Mitochondria are highly dynamic double‐membrane organelle that exist in almost all eukaryotic cells.^5^ The main function of mitochondria is to produce adenosine 5′‐ triphosphate (ATP) through oxidative phosphorylation (OXPHOS) to meet most of the energy requirements of cells.^6^, ^7^ Human mitochondrial DNA (mtDNA) is a circular molecule composed of 16,568 bases, encoding ribosomal RNA (rRNA), transfer RNA (tRNA) and the important components of mitochondrial electron transport chain (ETC).^8^ The number of mtDNA in the mitochondrial genome is called mtDNA copy number, which is specific in different types of tissue cells.^9^ El‐Hattab et al. have reported that the reduction of mtDNA copy number in cells can impair mitochondrial respiration and cause pathology as diverse as encephalopathy, neuropathy and ageing process.^10^ Ed Reznik et al. found that the expression of mitochondrial metabolic genes and the occurrence of some mutations will change the copy number of mtDNA and affect the function of mitochondria.^11^

The replacement loop region (D‐loop) of mtDNA is the only non‐coding region in the human mitochondrial genome, which contains about 1122bp, accounting for 6% of the total mtDNA molecular weight and involved in the regulation of mtDNA replication and transcription.^12^, ^13^ As the binding site of mtDNA and mitochondrial membrane, the D‐loop region rich in A and T bases is sensitive to oxidative stress, which is a high‐incidence region of mtDNA mutations. The ratio of base substitution is 6–8 times higher than that in other regions of the mitochondrial genome.^14^

A large number of replication and transcription factors are important to maintain the normal copy number of mtDNA and meet the energy requirements of cells.^15^ Mitochondrial transcription factor A (TFAM) encoded by the *TFAM* gene is the first discovered mitochondrial transcription factor, which is involved in mtDNA replication, transcription and mtDNA repair.^16^ Polymerase γ (Polγ) encoded by the *POLG* gene is the only DNA polymerase in human mitochondria, which participates in mtDNA replication and enhances the activity of enzymes by accelerating the polymerization rate and inhibiting the activity of exonucleases.^17^ Moreover, *POLG* gene mutation can lead to various mtDNA mutations and deletions as well as mtDNA depletion syndrome. The TWINKLE helicase encoded by *TWNK* gene is a mitochondrial 5'‐3’ helicase, which can bind to double‐stranded DNA (dsDNA), and dissociate it into single‐stranded DNA by breaking the hydrogen bond between the annealed nucleotide bases.^18^ Besides, TWINKLE helicase can cooperate with Polγ to participate in the replication of mtDNA.^19^ Peroxisome proliferator‐activated receptor γ coactivator‐1α (PGC‐1α), a transcriptional coactivator with multiple functional activities, plays a major role in the regulation of cellular energy homeostasis and mitochondrial oxidative metabolism.^20^ Studies have shown that PGC‐1α regulates the transcription of the mitochondrial genome by binding with NRF‐1 and NRF‐2 and activating the expression of the downstream genes *TFAM*, *TFB1 M* and *TFB2 M* that regulate the replication and transcription of mitochondrial DNA.^21^

However, there are few studies on the mechanism of variations in mtDNA copy number and its follow‐up effects in patients with IS. The specific mechanism of variations in mtDNA copy number caused by D‐loop mutation is still unclear.

Therefore, in the present study, firstly, mtDNA copy number levels of IS patients and normal controls were detected by quantitative real‐time PCR and the effect of mitochondrial genome D‐loop variation on mtDNA copy number of IS patients was explored. Then, by simulating the pathological process of cerebral ischaemia/reperfusion in IS, an oxygen‐glucose deprivation and reperfusion injury (OGD/R) model was constructed to seek genes that regulate mtDNA copy number changes in IS. Finally, the knockdown‐over‐expression cell model of *TFAM* gene was constructed, and the influence of variations in mtDNA copy number on mitochondrial function was explored by testing the enzyme activity of mitochondrial respiratory chain complex, mitochondrial membrane potential and ATP production.

## 2 MATERIALS AND METHODS

### 2.1 Study population

The case group consisted of 101 patients with IS (68 males and 33 females) with the average age of 53.02 ± 9.15 years who were diagnosed in the First Affiliated Hospital of Henan University of Traditional Chinese Medicine from April to August in 2018. The diagnosis of all IS patients was based on the IS diagnostic criteria revised by the fourth National Conference on Cerebrovascular Diseases. All IS patients were first onset and confirmed by clinical examination (physical signs, history, biochemical tests, CT/MRI and other ancillary diagnostic investigations), without a family history of cardiovascular disease, diabetes and hypertension. The control group consisted of 101 healthy individuals (61 males and 40 females) with an average age of 52.05 ± 5.87 years whose gender and age matched that of IS group from the same hospital during the same period, excluding those with a family history of cardiovascular disease, diabetes and hypertension. All the subjects were from the Henan Han population without blood relationship between them.

The study was permitted by the Ethics Committee on Human Research of Zhengzhou University. Written informed consent was obtained from all subjects. All experiments were performed in accordance with relevant guidelines and regulations.

### 2.2 Extraction and purification of peripheral blood DNA

2‐5mL of fasting peripheral venous blood was collected from subjects into tubes containing 2% EDTA‐K_2_ and stored in a refrigerator at −20℃ for subsequent assays. High‐purity genomic DNA was isolated from peripheral blood cells of subjects using the Blood DNA Extraction Kit (TIANGEN). Concentration and quality of DNA were detected by NanoDrop2000 (Thermo Fisher).

### 2.3 Detection of mtDNA copy number in population by Real‐time PCR

Real‐time quantitative PCR was performed on the mtDNA copy number of IS patients and normal subjects using the SYBR^®^ Premix Ex Taq^TM^ kit (Takara) and a QS5 quantitative PCR Systems (Thermo Fisher). The human DNA sequence of the *MT*‐*ND1* gene and *β*‐*globin* were obtained from the NCBI database, and the primers were designed and synthesized by Sangon Biotech (www.sangon.com/). Primers for *MT*‐*ND1* were F: 5′‐CCTAATGCTTACCGAACGA‐3′ and R: 5′‐GGGTGATGGTAGATGTGGC‐3′, Primers of *β*‐*globin* were F: 5′‐CTGCTGGTGGTCTACCCTTG‐3′ and R: 5′‐AGGCCATCACTAAAGGCACC‐3′. The qRT‐PCR reaction system and reaction conditions were set according to the instructions of Takara's SYBR^®^ Premix Ex Taq™ kit. The level of mtDNA copy number was represented by mtDNA/nDNA ratio, calculated by using the $2^{‐\Delta \Delta C_{t}}$ relative quantitative method.

### 2.4 Cell culture and oxygen‐glucose deprivation/re‐oxygenation (OGD/R) treatments

Rat adrenal medulla pheochromocytoma cell line PC12 was purchased from Shanghai Cell Bank of Chinese Academy of Sciences. PC12 cells were cultured in the prepared high‐glucose complete medium and maintained in an incubator with 5% CO_2_, 37°C, and 95% relative humidity. CCK8 Kit (Meilunbio) was used to detect the viability of cells by testing the absorbance at 450 nm by ELIAS (Thermo Fisher). An oxygen‐glucose deprivation/re‐oxygenation (OGD/R) treatment was performed on cultured PC12 cells. In short, when in the logarithmic growth phase, PC12 cells were cultured in serum‐free low‐glucose DMEM medium after washing with 1 × PBS and placed in an anaerobic incubator with 95% N_2_, 5% CO_2_ and 1% O_2_ for 2, 4, 8 and 12 h, respectively, and then CCK8 solution was used to detect cell viability of different time points, and the time point of cell oxygen and glucose deprivation treatment was determined finally. After the oxygen‐glucose deprivation, the cells were washed with 1 × PBS and cultured in high‐glucose DMEM medium of a CO_2_ cell incubator for 2, 4, 8, and 12 h respectively. Then the cell viability was detected by CCK8 solution at different time points, and the reperfusion treatment time point was determined. Cells in different time groups were set with 3 multiple pores.

### 2.5 Extraction and purification of total cell DNA and RNA, RNA reverse transcription

Primarily, treat adherent PC12 cells as cell suspension, transfer to 1.5 ml centrifuge tube, centrifuge at 13572 *g* for 1 min, discard supernatant. Then, high‐purity genomic DNA was isolated from PC12 cells using the TIANAMP Genomic DNA Extraction Kit (TIANGEN), and RNA was extracted using TIANAMP Genomic RNA Extraction Kit (TIANGEN), according to the manufacturer's instructions. Concentration and quality of DNA and RNA were detected by Nanodrop2000 (Thermo Fisher). Total RNA was used as a template to synthesis cDNA by the Quantscript RT Kit (TIANGEN), and synthesized cDNA was stored at −20°C for subsequent experiments.

### 2.6 Detection of mtDNA copy number and mRNA expression of TFAM, TWNK, POLG and PGC‐1α in PC12 and HEK 293T cells by Real‐time PCR

Real‐time PCR analysis was performed for relative expression levels of *MT*‐*ND1* and *TFAM*, *TWNK*, *POLG*, *PGC*‐*1α* mRNA on PC12 cell in OGD/R group and control group. The rat cDNA sequence of the *MT*‐*ND1* gene and *TFAM*, *TWNK*, *POLG*, *PGC*‐*1α* were obtained from NCBI database, and the primers were designed and synthesized by Sangon Biotech (www.sangon.com/). Primers for *MT*‐*ND1* were F: 5′‐GGATGAGCCTCAAACTCCAA‐3′ and R: 5′‐GGTCAGGCTGGCAGAAGTAA‐3′; Primers for *TFAM* were F: 5′‐GGGAATGTGGGGCGTGCTAAG‐3′ and R: 5′‐AGGCTGACAGGCGAGGGTATG‐3′; *TWNK* were F: 5′‐TCAGCCAAAGCCAGCCAAGAAG‐3′ and R: 5′‐TACCGTTTCCCAGGCCCAGTC‐3′; *POLG* were F: 5′‐ACCAGCACTTCCGCCTCCTG‐3′ and R: 5′‐ACCAGCACTTCCGCCTCCTG‐3′; *PGC*‐*1α* were F: 5′‐TGACCCTCCTCACACCAAACCC‐3′ and R: 5′‐TTGCGACTGCGGTTGTGTATGG‐3′; Primers of *β*‐*actin* were F: 5′‐TGGCACCCAGCACAATGAA −3′and R: 5′‐ACCAGCACTTCCGCCTCCTG‐3′. Nuclear‐encoded housekeeping gene *β*‐*actin* was used as an internal control. The relative expression of gene was calculated using the $2^{‐\Delta \Delta C_{t}}$ relative quantitative method.

### 2.7 Plasmids transfection in HEK293T cells

Human embryonic kidney cells HEK 293T was purchased from Shanghai Cell Bank of Chinese Academy of Sciences. GV102 knockout plasmid vector and GV141 overexpressed plasmid vector were purchased from Genechem (Shanghai). HEK 293T cells were transfected with knockdown and overexpressed TFAM plasmid by Simple‐fect transfection reagent (Signaling Dawn Biotech) respectively. The recombinant product through the connection of the linear vector and the target gene fragment had been identified and sequenced by PCR. The results of the sequencing and the alignment of the target gene sequence were completely consistent. E.Z.N.Z Plasmid Mini kit (Omega Biotek) was used for plasmid extraction according to manufacturer's instruction. The concentration of plasmid DNA was measured by Nanodrop2000 (Thermo Fisher), then the extracted plasmid was stored at −20°C for subsequent experiments.

### 2.8 Western blot

Total protein extracted from HEK 293T cells was, respectively, loaded onto a polyacrylamide gel and analysed by Western blot, with an equal amount (20 μg) per lane. Blots were subsequently detected with the following antibodies: HSP60 (D6F1) XP^®^ Rabbit mAb (Cell Signaling Technology), Anti‐TFAM Rabbit pAb (ZEN BIO), Goat anti‐Rabbit IgG (H&L; ZEN BIO), Mouse Anti‐β actin mAb (ZSGB‐BIO), Goat Anti‐Mouse IgG Secondary Antibody (Sino Biological). Protein bands were detected using an ECL analysis system.

### 2.9 Detection of mitochondrial respiratory chain complex activity, mitochondrial membrane potential, and mitochondrial ATP level

The activity of the mitochondrial respiratory chain complex was, respectively, measured using the respiratory chain complex activity detection kit (Solarbio). In brief, mitochondria were first extracted from HEK 293T cells transfected with plasmids for subsequent determination of complex activity and concentration, then the prepared reagents and working solution were mixed with the sample to determine the absorbance by the full wavelength microplate reader (Thermo Fisher), the activity of complexes was calculated according to the formulas in the manufacturer's instructions.

The mitochondrial membrane potential detection kit (JC‐1) (Beyotime Biotechnology, Shanghai, China) was used in the detection of mitochondrial membrane potential. Briefly, cells were resuspended in culture medium and adjusted to an appropriate density, and then stained with JC‐1 fluorescent probe for 20 min at 37°C. Finally, resuspended cells were analysed by FACS Celesta analytical flow cytometry (BD).

ATP level was measured with an ATP assay kit (Beyotime Biotechnology, Shanghai, China) according to the manufacturer's instructions. In brief, the lysate of HEK 293T cells transfected with plasmids was centrifuged at 4°C at 12,000 g for 5 min. Then 100 μl ATP detection working solution and 50 μl supernatant were mixed, and subsequently, Centro LB960 microplate luminescence detector (Berthold, Germany) was used for the detection of luminescence. The level of ATP was calculated according to the standard curve.

### 2.10 Statistical analysis

All statistical analyses were performed using SPSS21.0 software. The experimental data was evaluated for normality and homogeneity of variance. The quantitative data was analysed by Independent Sample *t*‐test or one‐way ANOVA, which were expressed as Mean ±SD. The Student's *t*‐test or rank sum test was used to analyse differences of the biochemical indexes and the D‐loop region mutation between IS group and control group. The copy numbers of mtDNA for IS group and control group also accepted the test by the Student's *t*‐test. The correlation between oxidative stress index and mtDNA copy number was analysed by Pearson correlation analysis or Spearman correlation analysis. The value of *p* < 0.05 indicated that the difference was statistically significant.

## 3 RESULTS

### 3.1 Comparison of variations in mtDNA copy number in peripheral blood between IS group and control group

**FIGURE 1:**
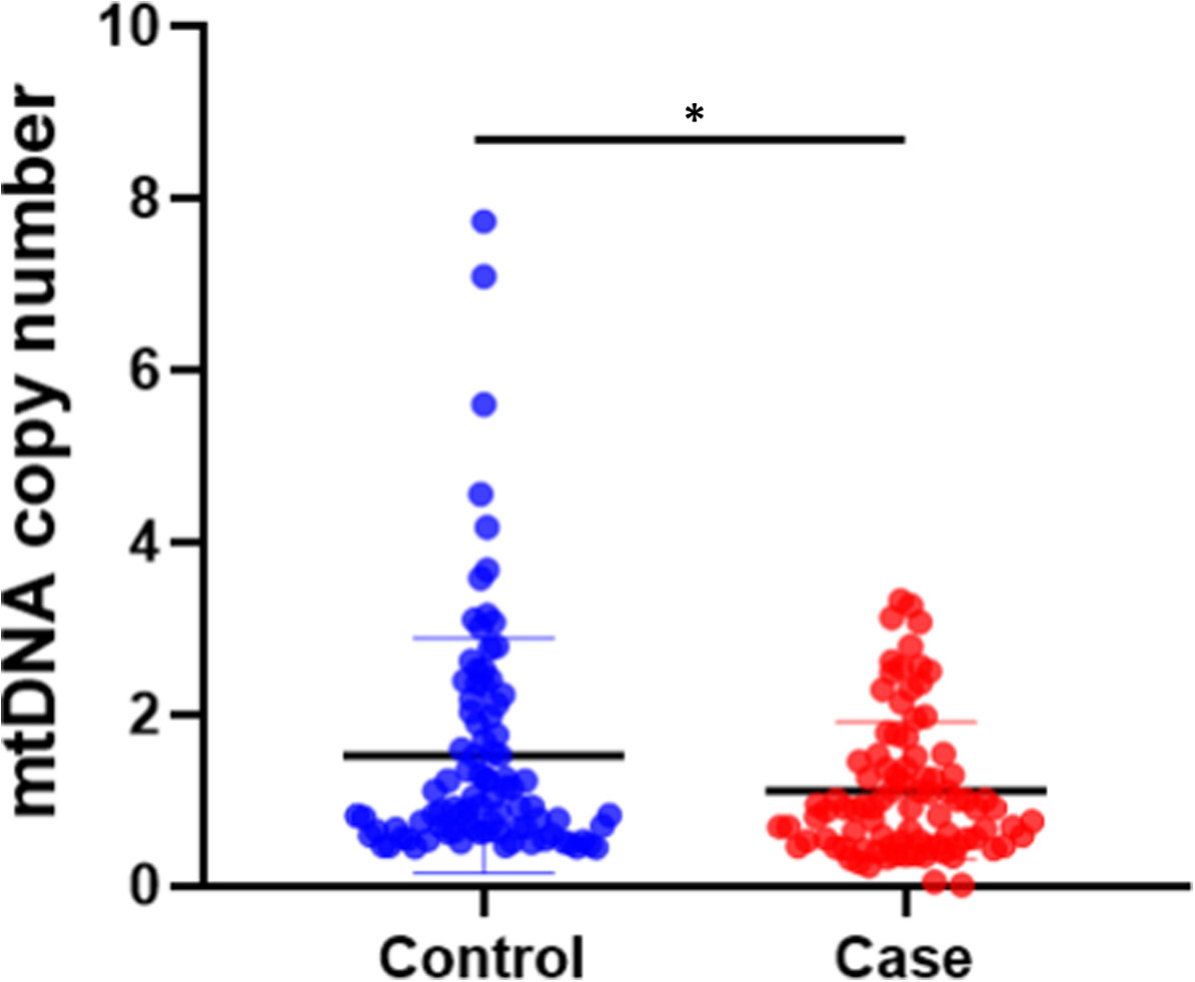
MtDNA copy number level in peripheral blood of IS group and control group (*p < 0.05)

The clinical information of the study populations and the comparison results of the clinical data were summarized in Table S1. The level of mtDNA copy number in the IS group (1.11±0.8) was significantly lower than that in control group (1.52 ± 1.37; *p* < 0.05; Figure 1). Moreover, the results of gender stratification showed that the mtDNA copy number of IS patients was significantly lower than that of controls (*p* < 0.05) in the male group, but there was no statistical difference between the IS cases and the controls in the female group (*p* < 0.05), as shown in Table S2 and Figure S1; while age stratification results indicated that the mtDNA copy number of IS patients was significantly lower than that of the controls (*p* < 0.05) in the over‐50‐year‐old group, but there was no statistical difference between the IS group and the control group under the age of 50 years (*p* < 0.05), as shown in Table S3 and Figure S2.

### 3.2 The effect of mitochondrial genome D‐loop mutation on mtDNA copy number

**FIGURE 2:**
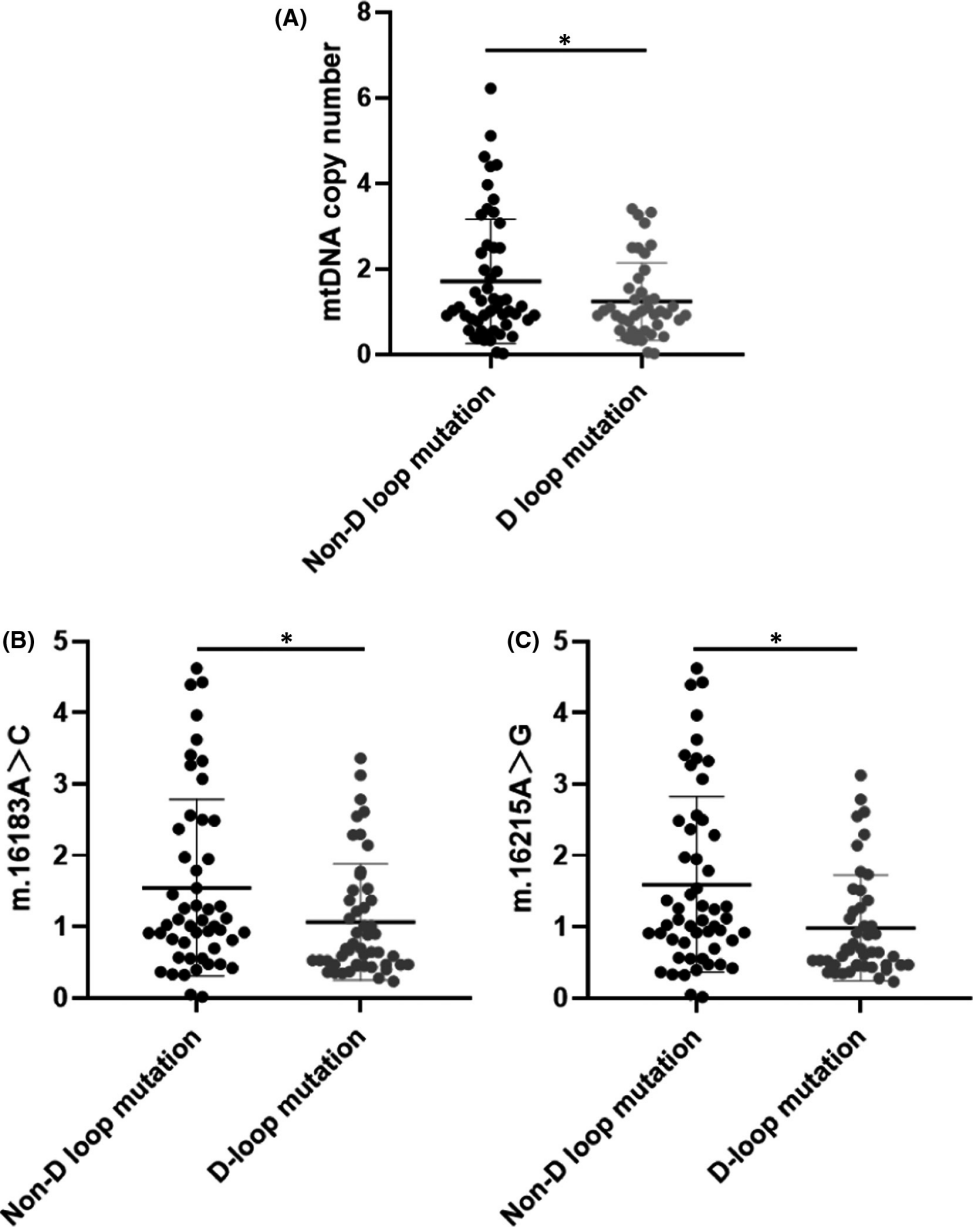
Effect of mitochondrial genome D‐loop mutation on mitochondrial DNA (mtDNA) copy number (A: The mtDNA copy number levels in IS group with and without D‐loop mutations, *p < 0.05; B–C: The mtDNA copy number levels in IS group with m.16215A > G and m.16355C > A mutations and without D‐loop mutation, *p < 0.05)

In our previous study, 7 mutation sites located in the D‐loop region of the mitochondrial genome were detected in both the IS group and the control group, of which there was a significant difference in the mutation proportion of the m.T195C site between the IS group and the control group (*p* < 0.01, Table S4). In this study, the mtDNA copy number of IS patients with and without the above 7 D‐loop mutation sites was statistically analysed. The results showed that the mtDNA copy number (1.08 ± 0.82) of IS patients with the above D‐loop mutation site was significantly lower than that of IS patients without the above D‐loop mutation site (1.55 ± 1.23; *p* < 0.05; Figure 2A). Furthermore, a single analysis of the 7 mutation sites showed that the mtDNA copy number of IS patients with m.16215A > G and m.16355C > A mutations (1.07 ± 0.82, 0.98 ± 0.74 respectively) was significantly lower than that of IS patients without D‐loop mutations (1.55 ± 1.24, 1.59 ± 1.23 respectively; *p* < 0.05). The results were shown in Figure 2B‐C.

### 3.3 Detection of mtDNA copy number in OGD/R cells model

**FIGURE 3:**
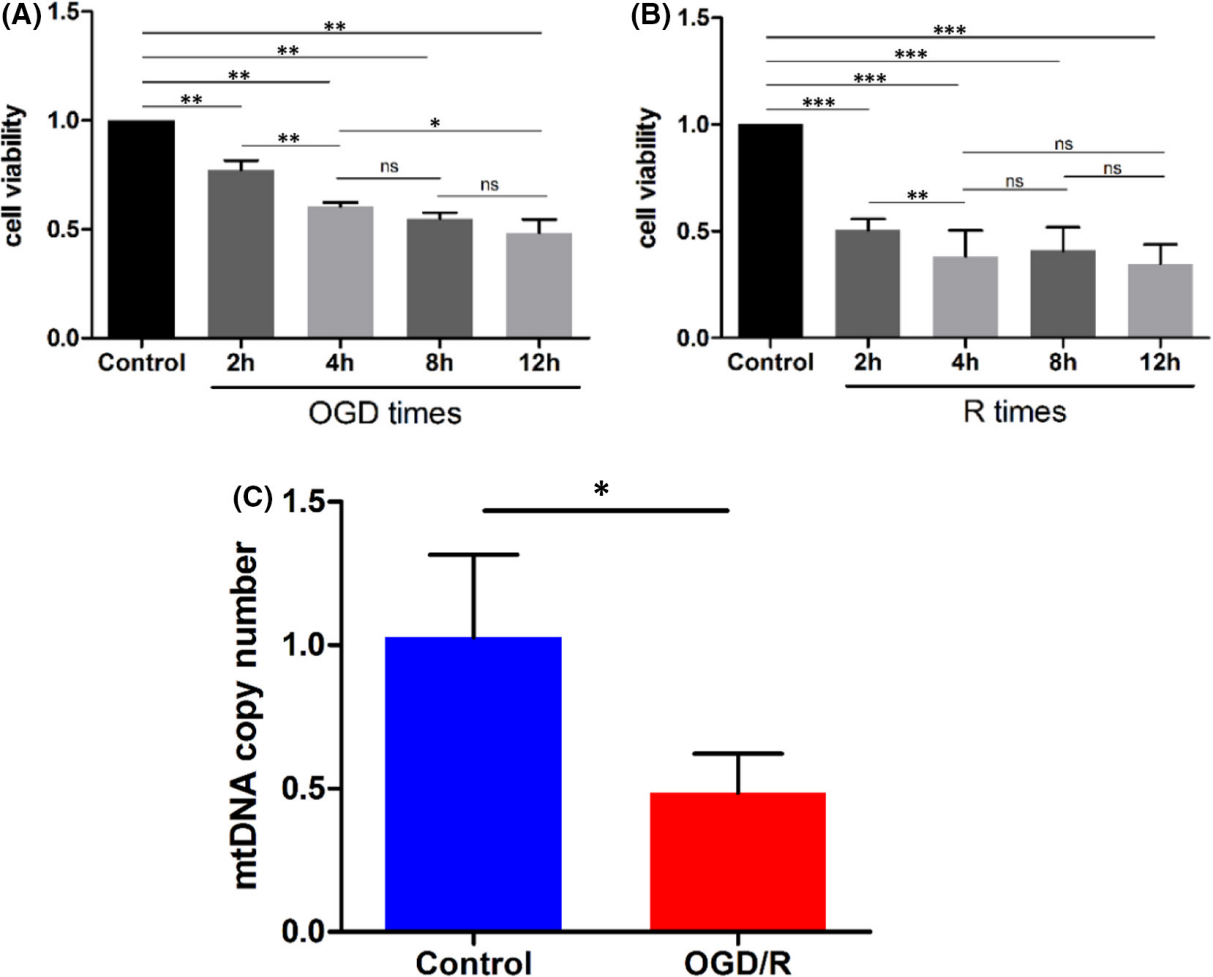
Detection of mtDNA copy number in OGD/R cells model (A‐B: Cell viability detection at different time points after OGD/R, *p < 0.05, **p < 0.01, ***p < 0.001, ns: nonsignificant; C: The mtDNA copy number levels of OGD/R group and control group, *p < 0.05)

The results of cell viability test at different time points after OGD/R treatment showed that the cell viability decreased to 60% after 4 h of oxygen‐glucose deprivation, and the cell viability continued to decrease with a longer treat time, but it was easy to cause irreversible damage to the cell. When 4 h for oxygen‐glucose deprivation was chosen, the cell viability was reduced to the lowest after 4 h of reperfusion; therefore, OGD 4h/R 4 h is the optimal modelling time for oxygen‐glucose deprivation/reperfusion model (OGD/R). The results were shown in Figure 3A–B. The mtDNA copy number of cells in the OGD/R group (0.49±0.14) was significantly lower than that in the control group (1.03 ± 0.29; *p* < 0.05), which was consistent with the results of the population study (Figure 3C).

In addition, heat shock protein 60 (HSP60) is a chaperone protein necessary for the folding of mitochondrial proteins and the formation of multimeric complexes, which is an important indicator for changes in mitochondrial expression. The results showed there was no difference in HSP60 protein expression between the OGD/R group and the control group (*p* > 0.05), which indicated that the mitochondria expression in the cells did not change after OGD/R injury. The results are shown in Figure S3.

### 3.4 Research on genes regulating mtDNA copy number in OGD/R cells model

**FIGURE 4:**
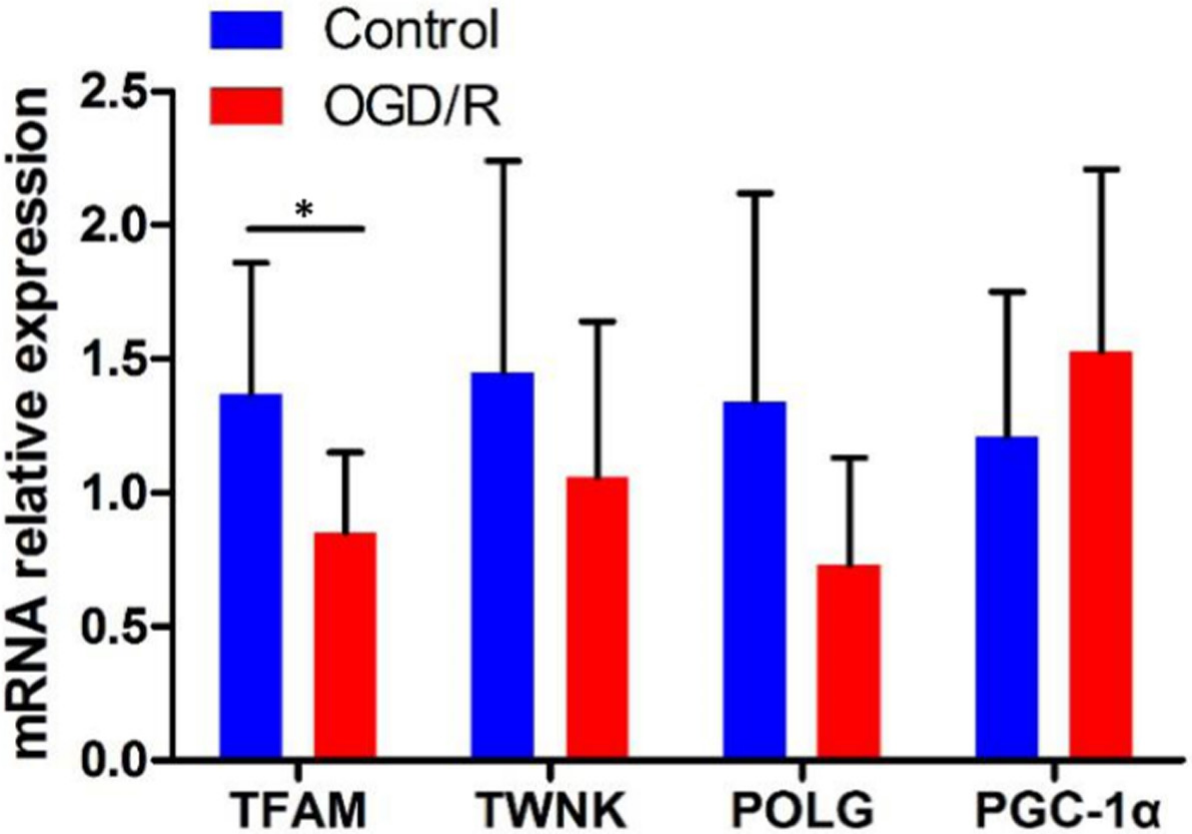
Relative expression levels of *TFAM*, *TWNK*, *POLG*, *PGC*‐*1α* mRNA in OGD/R group and control group, *p < 0.05

To explore genes that regulate mtDNA copy number of cells in the OGD/R group, real‐time fluorescence quantitative PCR was performed for relative mRNA expression levels of *TFAM*, *TWNK*, *POLG*, *PGC*‐*1α*. The results showed that the relative mRNA expression level of *TFAM* gene in the OGD/R group was significantly lower than the control group (*p* < 0.05), and the relative mRNA expression levels of *TWNK*, *POLG* and *PGC*‐*1α* gene were not statistically significant between the two groups (*p *> 0.05), which suggested the *TFAM* gene might regulate variations in mtDNA copy number in OGD/R cells model. The results were shown in Table 1 and Figure 4.

**TABLE 1:** Relative expression levels of *TFAM*, *TWNK*, *POLG*, *PGC*‐*1α* mRNA

Gene	Control group	OGD/R group	*p* value
*TFAM*	1.37 ± 0.49	0.85 ± 0.30	0.035*
*TWNK*	1.45 ± 0.79	1.06 ± 0.58	0.315
*POLG*	1.34 ± 0.78	0.73 ± 0.40	0.051
*PGC−1α*	1.21 ± 0.54	1.53 ± 0.68	0.490

*p < 0.05, and the difference is statistically significant.

### 3.5 Effect of variations in mtDNA copy number on mitochondrial function

#### 3.5.1 Construction of TFAM gene knockdown‐over‐expression cell model

**FIGURE 5:**
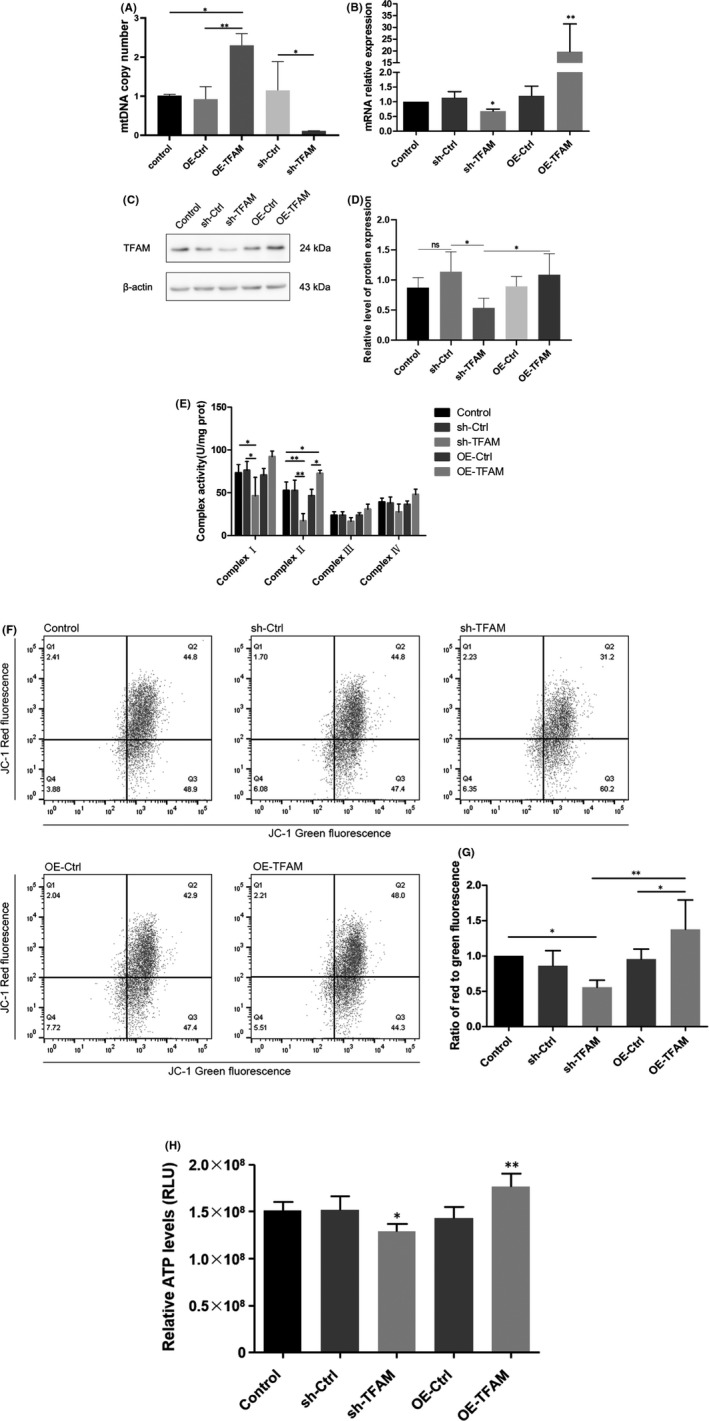
Effect of variations in mtDNA copy number on mitochondrial function (A‐D: verification of variations in mtDNA copy number cell model by qRT‐PCR and by Western Blot, *p < 0.05, **p < 0.01; E: The levels of mitochondrial respiratory chain complex activity, *p < 0.05, **p < 0.01; F–G: The levels of mitochondrial membrane potential, *p < 0.05, **p < 0.01; H: The levels of mitochondrial ATP generation, *p < 0.05, **p < 0.01)

HEK 293T cells were, respectively, transfected with *TFAM* gene knockdown and over‐expression plasmids to construct cell models of mtDNA copy number reduction and increase. The effect of plasmid construction and transfection efficiency was detected by qRT‐PCR and Western blot. After the *TFAM* gene was knocked down and overexpressed in HEK 293T cells, the expression levels of *TFAM* gene and protein were significantly decreased or increased in different groups (*p* < 0.05), and the cell model of variations in mtDNA copy number was successfully constructed. The results were shown in Figure 5A–D

### 3.6 Effect of variations in mtDNA copy number on mitochondrial respiratory chain complexes

In HEK 293T cells transfected with knockdown *TFAM* plasmid (sh‐TFAM), the activity of mitochondrial respiratory chain complex I and II decreased significantly (*p* < 0.05); while after transfection of HEK 293T cells with overexpressed *TFAM* plasmid (OE‐TFAM), the activity of the complex II was remarkably increased (*p* < 0.05), and the activity of the complex I showed an increasing trend which yet had no statistical difference. Besides, although the activity of the complex III and IV demonstrated a trend of decrease and increase in sh‐TFAM group and OE‐TFAM group, there was no significant difference between the experimental groups (*p* > 0.05). The results were shown in Figure 5E.

### 3.7 Effect of variations in mtDNA copy number on mitochondrial membrane potential

Compared with the control group, the mitochondrial membrane potential of HEK 293T cells significantly decreased in the sh‐TFAM group (*p* < 0.05); while the mitochondrial membrane potential of HEK 293T cells in the OE‐TFAM group was dramatically higher than that in the OE‐Ctrl group (*p* < 0.05; Figure 5F‐G).

### 3.8 Effect of variations in mtDNA copy number on mitochondrial ATP production

The ATP generation level of HEK 293T cells transfected in the sh‐TFAM group was significantly lower than that in the control group and sh‐Ctrl group (*p* < 0.05), and compared with the control group and the OE‐Ctrl group, the ATP production level of the OE‐TFAM group was markedly increased (*p *< 0.01; Figure 5H).

## 4 DISCUSSION

MtDNA copy number is the number of mtDNA in the mitochondrial genome. Each contains 2–10 mtDNA copies. The copy number of mtDNA in each somatic cell of normal people is about 10^3^–10^4^, which is specific in different types of tissue cells.^22^ The mtDNA copy number of tissues and organs with high energy dependence is relatively higher than that of tissues and organs with low energy dependence.^23^ The change of mtDNA copy number can affect the function of mitochondria and play an important role in the occurrence and development of diseases.

Our results showed that the mtDNA copy number level of the IS group was significantly lower than that of the normal control group (*p* < 0.05). In the gender stratification analysis, it was found that the mtDNA copy number of the male IS group was significantly lower than that of the male control group (*p* < 0.05), but there was no significant difference between the female IS group and the female control group (*p* > 0.05). In the age stratification analysis, the mtDNA copy number in the IS patients over 50 years old was significantly lower than that in the normal control group (*p* < 0.05), but there was no significant difference between the IS group and the control group under 50 years old (*p* > 0.05). Lien et al. found that the mtDNA copy number of IS patients was significantly lower than that of the normal population.^24^ Ashar et al. also reported that the copy number of mtDNA in stroke patients was significantly reduced in large samples of cardiovascular disease, and the copy number of mtDNA was negatively correlated with the incidence of stroke.^25^ Song et al. conducted OCSP typing of stroke patients and found that mtDNA copy number was only related to all‐cause mortality of lacunar infarction stroke. Different stroke subtypes had different susceptibility to variations in mtDNA copy number. MtDNA copy number may be an independent risk factor for lacunar infarction stroke.^26^ Those results were consistent with our findings. The results of the study showed that the mtDNA copy number of IS patients was significantly lower than that of normal people. Male IS patients and IS patients aged over 50 years were susceptible to variations in mtDNA copy number.

In our previous study, through whole mitochondrial genome sequencing and multiple snapshot technology, 7 mtSNPs (m.195T > C, m.311C > T, m 16164A > G, m.16183A > C, m.16215A > G, m.16335C > A and m.16390G > A) located in the D‐loop region of the mitochondrial genome and detected simultaneously in IS group and control group were selected respectively. In order to explore the effect of mitochondrial D‐loop mutation on mtDNA copy number of IS patients, we compared the mtDNA copy number of IS patients with 7 D‐loop mutation sites and IS patients without D‐loop mutation sites. After that, we compared the mtDNA copy numbers of the patients with single mutation site and those without D‐loop mutation. The results showed that the mtDNA copy number of IS patients with D‐loop mutation sites was significantly lower than that of IS patients without D‐loop mutation sites (*p* < 0.05). The mtDNA copy number of IS patients with m.a16215g mutation and m.c16355a mutation was significantly lower than that of IS patients without D‐loop mutation (*p *< 0.05). Zhang et al. found that mutations in the D‐loop region in gastric cancer patients may lead to the increase of mitochondrial genome replication cycle, decrease of mtDNA copy number or change of mitochondrial gene expression.^27^ Grady et al. reported that mtDNA copy number in blood was low in patients with MTL‐TL1 M. A3243G mutation and mtDNA copy number and M. A3243G heterogeneity showed weak correlation, suggesting that single mutation of mitochondrial genome may affect variations in mtDNA copy number.^28^ C. Yin et al.'s research showed that the proliferation, invasion and metastasis of HCC cells with D‐loop mutation were significantly higher than those without D‐loop mutation Mutant hepatoma cells.^29^ Our results showed that mutations in the D‐loop region m.16215A > G and m.16355C > A sites can affect the variation of mtDNA copy number. We speculated that the mtSNPs in the D‐loop region may lead to the increase of mtDNA replication cycle, affect mtDNA replication and transcription, and then cause variations in mtDNA copy number in patients with IS. However, the mechanism of the variation of mtDNA copy number caused by D‐loop mutation needs further study.

Ischaemia/reperfusion (IR) injury is the basis of IS. Oxygen glucose deprivation and reperfusion (OGD/R) cell model is a common model for in vitro study of IS.^30^ Cells are cultured in low‐glucose serum‐free medium and anaerobic conditions for a period of time, and then the cells are replaced with glucose‐containing medium and normal oxygen concentration to stimulate the pathophysiological process of tissue or cell IR damage. In the OGD/R cell model, the mtDNA copy number of OGD/R group was significantly lower than that of control group (*p* < 0.05), indicating that there were variations in mtDNA copy number in the OGD/R model. The results of OGD/R model were consistent with the results of variations in mtDNA copy number in the population.

A large number of replication and transcription factors such as *TFAM*, *Polγ*, *TWNK*, *PGC*‐*lα*,which play an important role in mtDNA replication, transcription and mtDNA repair, and participate in the regulation of mtDNA copy number, are important guarantee to maintain the normal mtDNA copy number and meet the energy requirements of the cell.^31^, ^32^ Therefore, real‐time fluorescence quantitative PCR was used to detect the expression of *TFAM*, *Polγ*, *TWNK* and *PGC*‐*lα* genes in the OGD/R model to explore the molecular mechanism of regulating mtDNA copy number. The results showed that the relative expression of *TFAM* gene mRNA in the OGD/R treatment group was significantly lower than that in the control group (*p* < 0.05), while the relative expression of *TWNK*, *POLG*, *PGC*‐*1α* gene was not statistically different between the two groups (*p* > 0.05). It was suggested that the copy number variation of mtDNA might be regulated by *TFAM* in the OGD/R cell model. Clay et al. showed that over‐expression of *TFAM* in the light chain promoter region (LSP) and heavy chain promoter region 1 (HSP1) of mitochondrial can enhance the transcription of LSP and HSP1 DNA. *TFAM* is essential for the regulation of mtDNA copy number.^22^ Filograna et al. reported that knocking down the *TFAM* gene in transgenic mice would lead to the loss of mtDNA in mice.^33^ On the other hand, Ekstrand et al. reported that over‐expression of *TFAM* led to an increase in mtDNA copy number.^34^ These results suggested that the variations in mtDNA copy number in OGD/R model might be due to the decrease of *TFAM* mRNA expression and abnormal function of *TFAM* gene after OGD/R injury.

Mitochondria is the main place where cells undergo oxidative phosphorylation and synthesize ATP and are the centre of energy conversion and output in the process of cell metabolism.^9^ It has been reported that variations in mtDNA copy number can affect the intracellular energy supply, reduce the level of oxidation and mitochondrial dysfunction, and then lead to apoptosis. We have confirmed that variations in mtDNA copy number in the OGD/R cell model were mainly regulated by the *TFAM* gene. Therefore, *TFAM* knockdown and over‐expression plasmids were constructed and transfected into HEK 293T cells and cell model of variations in mtDNA copy number was established in vitro. qRT‐PCR and Western Blot were used to detect the plasmid construction effect and transfection efficiency.

The respiratory chain of mitochondria is composed of complex I, II, III and IV, which make the electron from high to low according to redox potential low transfer, energy release step by step.^35^ The activity of respiratory chain complex directly affects the oxidative phosphorylation function of mitochondria.^36^ Our results showed a reduction in mtDNA copy number would cause a decrease in the activity of the mitochondrial respiratory chain complex, and an increase in mtDNA copy number would rescue the function of the complex. Filograna et al. have constructed a *TFAM* gene knockout and over‐expression transgenic mouse model and found that in the *TFAM* gene knockout group, the copy number of mtDNA in mice was reduced, and the results of COX and SDH activity staining showed abnormal mitochondrial respiratory chain function and decreased tRNA^Ala^ protein expression, and the translation of mitochondria was impaired.^33^ In the *TFAM* gene over‐expression group, the copy number of mice mtDNA increased, the abnormal function of the mouse respiratory chain was rescued, the expression of tRNA^Ala^ protein increased, and mitochondrial translation was restored.

The energy released by the mitochondrial respiratory chain during electron transfer is used to drive protons from the mitochondrial matrix to the membrane space.^37^ Due to the high impermeability of the inner membrane to H^+^, a potential gradient across the mitochondrial inner membrane is created, resulting in a negative internal membrane potential.^38^ Normal membrane potential is necessary to maintain mitochondrial oxidative phosphorylation function. We used JC‐1 fluorescent probe to detect the changes in mitochondrial membrane potential. The results showed that compared with the blank control group, the mitochondrial membrane potential of the sh‐TFAM transfected group was significantly reduced (*p* < 0.05), the OE‐TFAM transfected group was increased significantly when compared with OE‐Ctrl group (*p *< 0.05). Therefore, increasing the copy number of mtDNA can rescue the decrease of mitochondrial membrane potential. Fiebig et al. reported that genetic inhibition of mitochondrial transcription by conditional deletion of *TFAM* led to dysfunctional ETC and OXPHOS activity, as indicated by aberrant mitochondrial swelling in astrocytes.^39^

The respiratory chain of mitochondria is coupled with the phosphorylation of ADP in the process of electron transfer. Under the action of ATP synthase, ADP and 1‐molecule phosphate are combined to form ATP, providing energy for life activities.^40^ Our results showed that after sh‐TFAM transfection, mtDNA copy number decreased, ATP production level was significantly decreased compared with blank control group and Sh‐Ctrl negative control group (*p* < 0.05). The ATP production level of the OE‐TFAM transfected group was significantly higher than that of blank control group and OE‐ctrl negative control group (*p* < 0.01), indicating that the increase of mtDNA copy number can promote intracellular ATP production. Hori A et al. found that mtDNA encodes proteins that are essential for cellular ATP production.^41^

The present study includes several limitations. Firstly, further research of specific mechanism of variations in mtDNA copy number caused by mutations in the D‐loop region is needed. Secondly, the mechanism of *TFAM* gene function damage after OGD/R injury is still unclear. Thirdly, the mechanism of mitochondrial copy number regulation of mitochondrial function needs further study.

**FIGURE 6:**
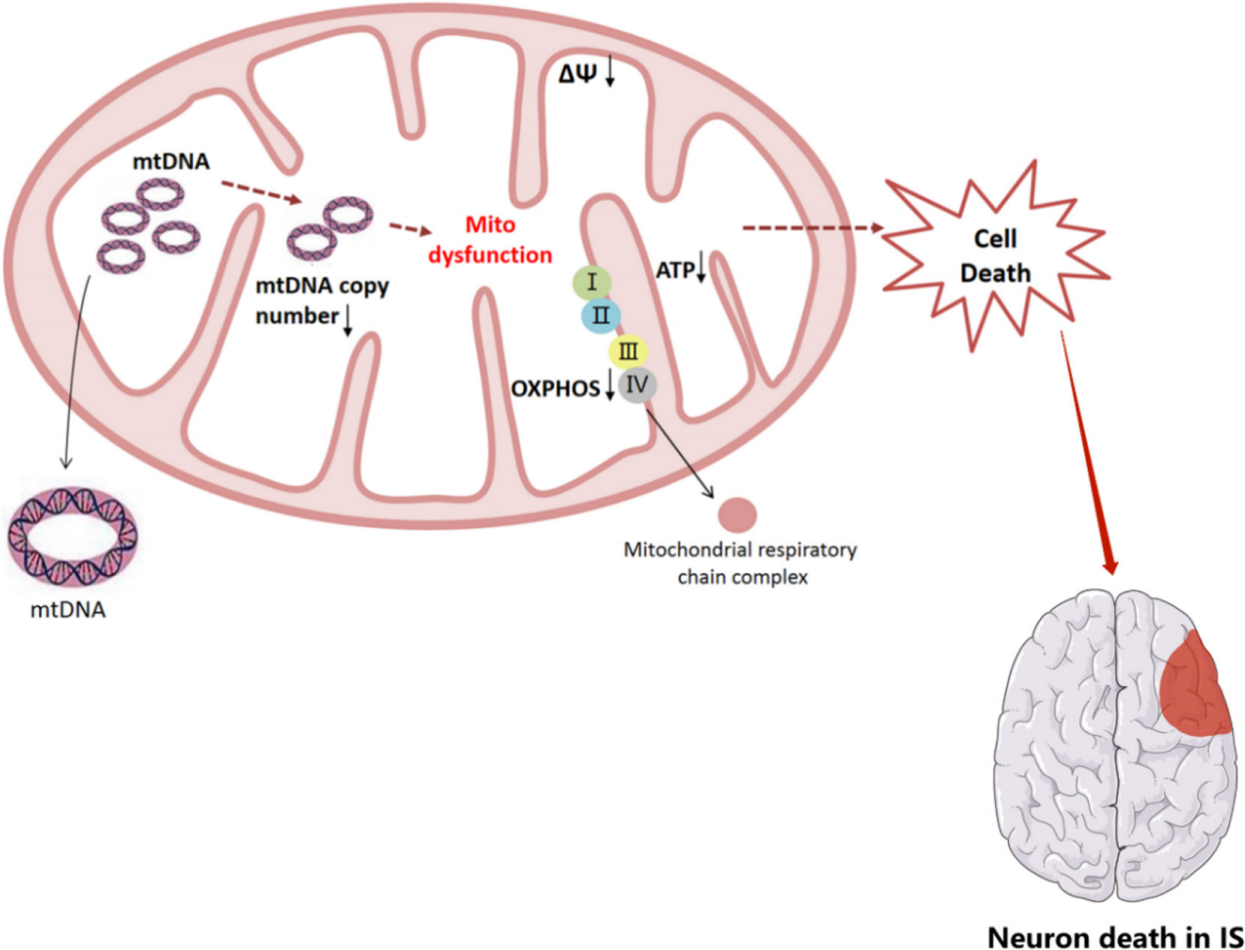
Influence of mtDNA copy number mutation on mitochondrial function

In brief, the study demonstrated that mitochondrial D‐loop mutation and *TFAM* gene dysfunction can cause the decrease of mtDNA copy number, resulting in the decrease of mitochondrial respiratory chain complex activity, membrane potential and ATP production, thus affecting the mitochondrial metabolism and function of nerve cells and participating in the pathological damage mechanism of IS. In addition, two new mutations, m.16215 G > A and m.16355 C > A, which affect variations in mtDNA copy number, were found in the D‐loop region and provide a new theoretical basis for variations in mtDNA copy number (Figure 6).

## CONFLICT OF INTEREST

The authors declare that they have no conflicts of interest with the contents of this article.

## AUTHOR CONTRIBUTIONS

**Zhaojing Zhang:** Investigation (lead). **Dongzhi Yang:** Supervision (equal). **Baixue Zhou:** Investigation (supporting). **Yingying Luan:** Investigation (supporting). **Qihui Yao:** Data curation (lead). **Yang Liu:** Formal analysis (equal). **Shangdong Yang:** Methodology (equal). **Jing Jia:** Supervision (equal). **Yan Xu:** Supervision (equal). **Xiaoshuai Bie:** Writing – original draft (equal). **Yuanli Wang:** Writing – original draft (equal). **Zhihao Li:** Writing – original draft (equal). **Aifan Li:** Methodology (equal). **Hong Zheng:** Supervision (equal). **Ying He:** Supervision (lead).

## Supporting information

Supplementary Material

## Data Availability

The data that support the findings of this study are available from the corresponding author upon reasonable request.
