# Supplementary material for: Decrease of MtDNA copy number affects mitochondrial function and involves in the pathological consequences of ischaemic stroke
Source: J Cell Mol Med. 2022 Jul 5;26(15):4157–68. doi: 10.1111/jcmm.17262 (PMC9344826; doi:10.1111/jcmm.17262)
Supplement: Supplementary file 1 — Supplementary Material [file JCMM-26-4157-s001.doc]

**Supplement:**

Table **S1.** Comparison of clinical data between control group and IS group

| Items | Control Group | IS Group | *p* value |
| --- | --- | --- | --- |
| Female/Male（case） | 61/40 | 68/33 | 0.305 |
| Age (year) | 52.05±5.87 | 53.02±9.15 | 0.371 |
| TC（mmol/L） | 4.25±0.75 | 4.23±1.16 | 0.880 |
| TG（mmol/L） | 1.13±0.37 | 1.55±0.83 | 0.000^***^ |
| HDL（mmol/L） | 1.25±0.21 | 1.09±0.34 | 0.000^***^ |
| LDL（mmol/L） | 2.85±0.46 | 2.53±0.89 | 0.000^***^ |
| GLU（mmol/L） | 4.84±0.56 | 6.04±2.51 | 0.001^**^ |
| UA（μmol/L） | 310.64±59.27 | 297.47±82.39 | 0.208 |

^**^*P* <0.01, ^***^*P* <0.001, *and the difference is statistically significant.*

Table **S2.** The mtDNA copy number levels of different sexes in IS group and control group

| Gender | | Control Group | | IS Group | | *p* value | |
| --- | --- | --- | --- | --- | --- | --- | --- |
| Male | | 1.47±1.26 | | 1.09±0.83 | | 0.047^*^ | |
| Female | 1.62±1.54 | | 1.34±0.97 | | 0.754 | |  |

^*^*P* <0.05，*and the difference is statistically significant.*


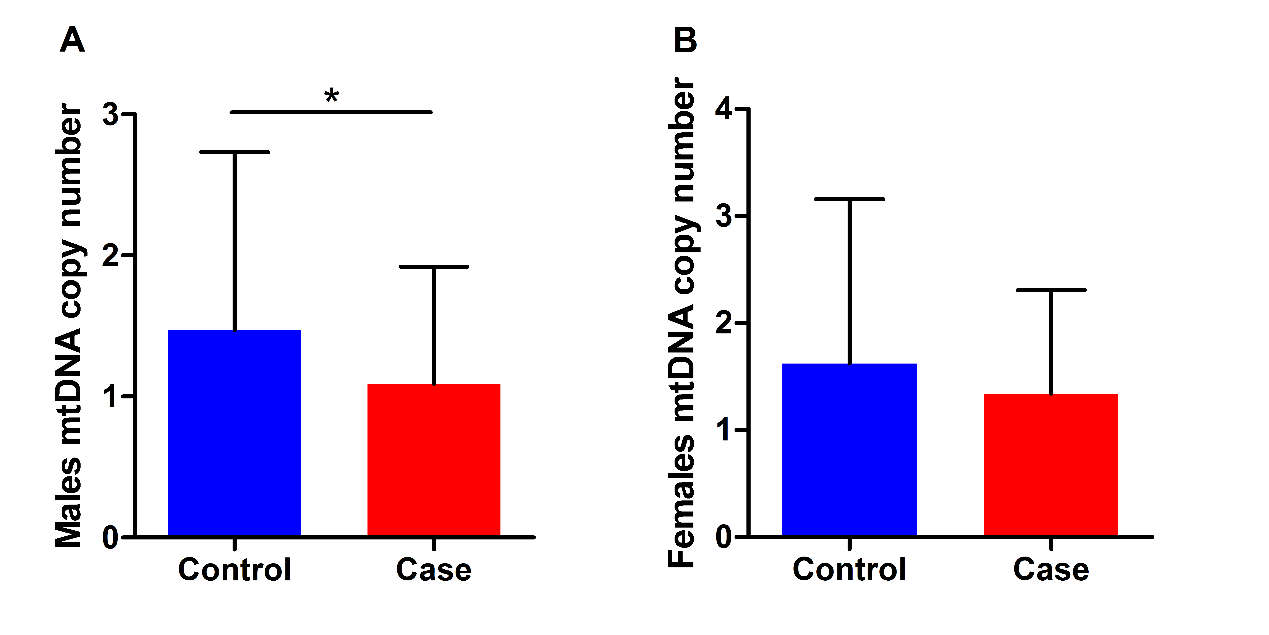
**Fig S1.** The results of gender stratification (**A**: male, **B**: female), ^*^*P* <0.05.

**Table S3****. The mtDNA copy number level of different age groups in IS group and control group**

| Age(year) | | Control Group | | IS Group | | *p* value | |
| --- | --- | --- | --- | --- | --- | --- | --- |
| ≤50 | | 1.30±0.98 | | 1.34±0.96 | | 0.662 | |
| ＞50 | 1.65±1.56 | | 1.07±0.79 | | 0.018^*^ | |  |

^*^*P* <0.05，*and the difference is statistically significant.*


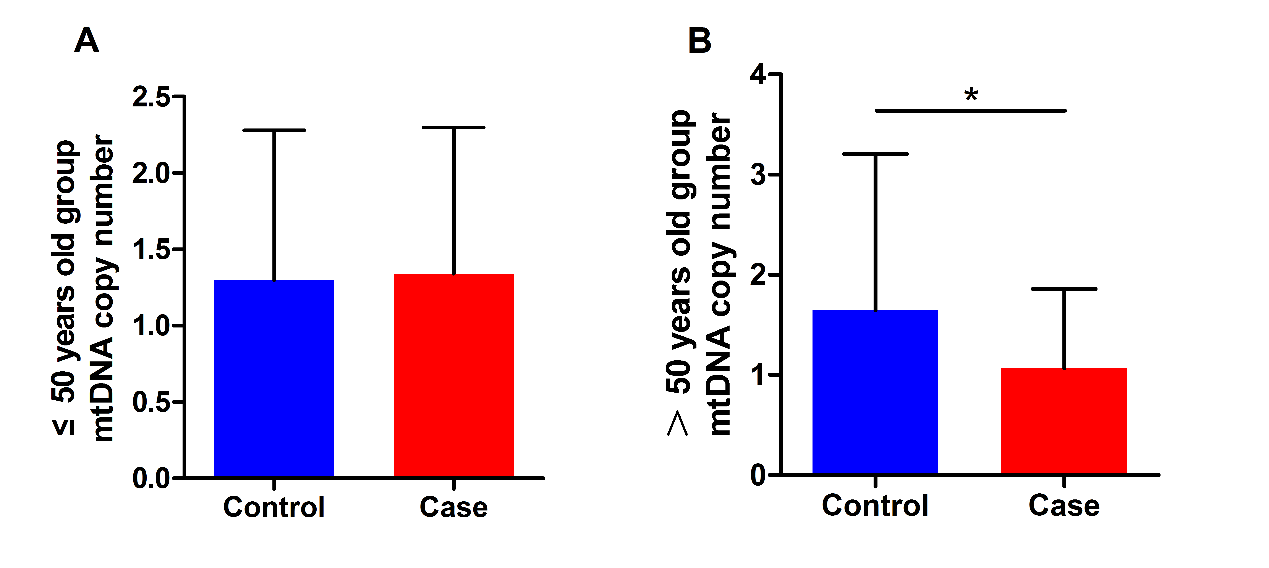


**Fig S2.** The results of age stratification (**A**:≤50, **B**:≥50), ^*^*P* <0.05.

Table **S4.** mtDNA mutation sites of D-loop region

| location | | gene | | Base  Change | | [proportion](../../../../E:/%25E6%259C%2589%25E9%2581%2593/Dict/8.5.3.0/resultui/html/index.html#/javascript:;) of Controls（%） | | [proportion](../../../../E:/%25E6%259C%2589%25E9%2581%2593/Dict/8.5.3.0/resultui/html/index.html#/javascript:;) of IS Group（%） | | *P* value | |
| --- | --- | --- | --- | --- | --- | --- | --- | --- | --- | --- | --- |
| 195 | | D-loop | | T-C | | 8.25 | | 3.53 | | 0.005^**^ | |
| 311 | D-loop | | C-T | | 6.5 | | 3.81 | | 0.086 | |  |
| 16164 | D-loop | | A-G | | 2.5 | | 1 | | 0.106 | |  |
| 16183 | D-loop | | A-C | | 67.75 | | 70.75 | | 0.358 | |  |
| 16215 | D-loop | | A-G | | 0.55 | | 1.97 | | 0.172 | |  |
| 16355 | D-loop | | C-A | | 2.25 | | 4.25 | | 0.111 | |  |
| 16390 | D-loop | | G-A | | 2.5 | | 2.51 | | 0.991 | |  |

^**^*P* <0.01, *and the difference is statistically significant.*


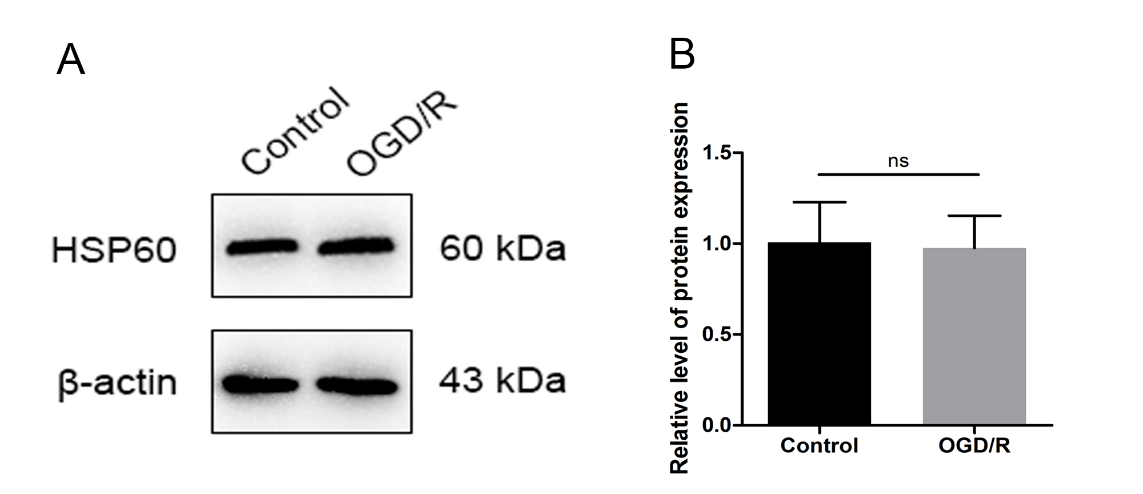


**Fig S3.** Detection of HSP60 protein expression (A: HSP60 expression by WB; B: the relative level of HSP60 expression, ns: nonsignificant)
